# CD44 fucosylation on bone marrow-derived mesenchymal stem cells enhances homing and promotes enteric nervous system remodeling in diabetic mice

**DOI:** 10.1186/s13578-021-00632-2

**Published:** 2021-06-30

**Authors:** Huiying Shi, Chen Jiang, Hailing Yao, Yurui Zhang, Qin Zhang, Xiaohua Hou, Rong Lin

**Affiliations:** 1 Department of Gastroenterology, Union Hospital, Tongji Medical College, Huazhong University of Science and Technology, Wuhan, 430022 China; 2 Department of Pathology, Union Hospital, Tongji Medical College, Huazhong University of Science and Technology, Wuhan, 430022 China

**Keywords:** Bone marrow-derived mesenchymal stem cells (BMSCs), Fucosylation modification, Diabetes, Gastrointestinal motility disorders, Enteric nervous system (ENS)

## Abstract

**Background:**

Diabetes can cause extensive enteric nervous system (ENS) injuries and gastrointestinal motility disorder. In developing possible treatments, researchers have engaged in tissue regeneration engineering with the very promising bone marrow-derived mesenchymal stem cells (BMSCs). However, BMSCs have poor homing ability to the targeted tissues after intravenous injection. Thus, we aimed to investigate whether enhancing the expression of E-selectin ligand on BMSCs could improve their homing ability and subsequently influence their role in ENS remodeling in diabetic mice.

**Methods:**

First, we constructed the fucosylation modification of CD44 on BMSCs through a fucosyltransferase VII (FTVII) system to generate a Hematopoietic Cell E-/L-selectin Ligand (HCELL) property, a fucosylated sialyllactosaminyl glycovariant of CD44 that potently binds E-selectin. Next, FTVII-modified and unmodified BMSCs labeled with green fluorescent protein (GFP) were injected into diabetic mice through the tail vein to compare their homing ability to the gastrointestinal tract and their effect on ENS remodeling, respectively. A bioluminescent imaging system was used to evaluate the homing ability of GFP-labeled BMSCs with and without FTVII modification, to the gastrointestinal tract. Gastrointestinal motility was assessed by gastrointestinal transient time, defecation frequency, stool water content and colon strips contractility. Immunofluorescence staining and western blotting were used to assess the expression levels of protein gene product 9.5 (PGP9.5), glial fibrillary acidic protein (GFAP) and glial cell line-derived neurotrophic factor (GDNF).

**Results:**

The FTVII-mediated α(1,3)-fucosylation modification of CD44 on BMSCs generated a HCELL property. Bioluminescent imaging assays showed that FTVII-modified BMSCs had enhanced homing ability to gastrointestinal tract, mainly to the colon, 24 h after injection through the tail vein. Compared with diabetic mice, FTVII-modified BMSCs significantly promoted the gastrointestinal motility and the ENS remodeling, including intestinal peristalsis (*P* < 0.05), increased feces excretion (*P* < 0.05) and the water content of the feces (*P* < 0.05), restored the spontaneous contraction of the colon (*P* < 0.05), and upregulated the protein expression levels of PGP9.5 (*P* < 0.01), GFAP (*P* < 0.001), and GDNF (*P* < 0.05), while unmodified BMSCs did not (*P* > 0.05).

**Conclusions:**

CD44 fucosylation modification on murine BMSCs promotes homing ability to the gastrointestinal tract and ENS remodeling in diabetic mice.

**Supplementary Information:**

The online version contains supplementary material available at 10.1186/s13578-021-00632-2.

## Introduction

The enteric nervous system (ENS), as an independent network of neurons and glial cells, is the most important direct factor in the regulation of gastrointestinal motility [1]. Diabetes can cause a wide range of ENS injuries [2], involving the stomach, small intestine, cecum and colon, including the size, number and degenerative diseases of intestinal neurons [3–8]. Clinical manifestations include diabetic gastroparesis, intestinal dysfunction (diarrhea, constipation, flatulence), etc. [2, 9]. However, there is no effective treatment for diabetic enteric neuropathy in clinical practice.

Bone marrow-derived mesenchymal stem cells (BMSCs) have been shown to promote nerve repair in a variety of nerve injury models. As reported, BMSCs pretreated with brain-derived neurotrophic factor combined with chondroitinase transplantation at the site of spinal cord transection injury in rats can significantly promote motor function recovery and spinal axon regeneration [10]. Similarly, in the rat model of sciatic nerve transection injury, Mohammadi R et al. found that the combination of local BMSCs grafted with pulsed electromagnetic fields can promote axon regeneration and recovery of transection nerve function [11]. Furthermore, in a diabetic mouse model, local intramuscular injection of BMSCs in the hind limbs can directly modulates peripheral neurogenesis, angiogenesis, and myelination to improve diabetic peripheral neuropathy [12].

BMSCs have become a cell therapy for a variety of diseases due to their safety, accessibility and immunity [13–15]. However, the poor directional homing ability of BMSCs in vivo prevent their colonization in local tissues after systemic intravenous administration, and thus fails to further play an effective repair role, which greatly limits their application and efficacy in cell therapy [16, 17].

Previous studies have shown that fucosylation modification of CD44 molecules on the BMSCs can significantly improve their ability to homing to the injured site after systemic intravenous transplantation [18]. CD44 is a transmembrane glycoprotein widely distributes on the surface of MSCs, after its N-acetylglucosamine is modified by α(1,3)-fucosylation, it can further form a sialyl-Lewis X (sLe^X^) structure, which can potently bind to E-selectin [19, 20]. CD44 forms a phenotype with a sLe^X^ structure-containing phenotype known as the hematopoietic cell E-/L-selectin ligand (HCELL). In the case of peripheral nerve injury, the expression of E-selectin, a ligand for HCELL, was significantly increased in the epineurium and endoneurium vascular endothelial cells [21]. Advanced studies showed that the binding of HCELL of BMSCs to the E-selectin on vascular endothelial cells can promote the homing ability of BMSCs to the injury site. Xiao et al. found that HCELL-positive BMSCs could migrate to the bone marrow of the model rabbits with tibial fractures through tail vein injection, while there was no obvious homing performance of unmodified BMSCs [22]. In a mouse model of renal ischemia–reperfusion injury, Chou et al. also found similar results, HCELL-positive BMSCs migrated to the damaged kidney in large numbers within 24 h after tail vein graft, while only a small amount of unmodified BMSCs could migrate to the damaged kidney [23]. However, whether the fucosylation-modified BMSCs can migrate to gastrointestinal tract in diabetic mice has not been reported.

This study, using the diabetic-induced ENS injury mice model, aimed to determine whether fucosylation-modified BMSCs have a better homing ability to the gastrointestinal tract and thus promoting the ENS remodeling and if so, to elucidate the mechanism.

## Materials and methods

### Animals

Wild-type and green fluorescent protein (GFP) transgenic C57BL/6 male mice (4-to 6-week-old, 18–22 g) purchased respectively from Beijing Vital River Laboratory Animal Technology Co., Ltd and Nanjing University Model Animal Research Center were used in this study. All animals were raised in the specific pathogen free grade laboratory of the Animal Experiment Center of Tongji Medical College, Huazhong University of Science and Technology under constant temperature (21–25 °C), humidity (50–60%) and photoperiods (12 h:12 h light–dark cycle). Animal study protocols were approved by the Animal Care Guidelines of Tongji medical college, Huazhong University of Science and Technology.

### BMSCs isolation and culture

BMSCs were isolated from the femur cavity of C57BL/6 mice using Dulbecco's Modified Eagle's Modified Medium (DMEM) (Gibco, NY, USA) under aseptic conditions. The cells were then cultured in a low-glucose DMEM medium with 10% fetal bovine serum (Gibco, NY, USA), incubated at 37 °C in a humidified incubator containing 5% CO_2_ for 24 h, and unattached hematopoietic cells were removed by changing the medium. Cells were digested with 0.25% trypsin (Gibco, NY, USA) when they were about 90% confluent. BMSCs at passage 6 to 8 were used for the following experiments. The identification and multidirectional differentiation ability of BMSCs were performed as our previous study described [24, 25].

### BMSCs exofucosylation

BMSCs were digested and washed twice with Ca^2+^- and Mg^2+^- free Hanks Balanced Salt Solution (HBSS) (Gibco, NY, USA). After centrifugation, the cell precipitates were treated with fucosyltransferase VII (FTVII) reaction buffer system, containing the 60 mU/mL FTVII (Cusabio, WuHan, China), 20 mM HEPES (Sigma, MissouriState, USA), 1% human serum albumin (HSA, Sigma, MissouriState, USA) and 1 mM GDP-fucose (Sigma, MissouriState, USA) in HBSS. The mixture was then incubated in a 5% CO_2_ incubator at 37℃ for 60 min, and mixed once every 15 min. The cells were then washed with HBSS containing 1% HSA and 20 mM HEPES and suspended in 1× phosphate-buffered saline (PBS) for later use. Cell viability was assessed by trypan blue staining.

### Evaluation of exofucosylation modification efficiency

The efficiency of exofucosylation modification on BMSCs was evaluated by the reactivity with mAb HECA452 (which recognizes canonical sialofucosylated E-selectin binding determinants (such as sialylated Lewis X (sLe^x^)) both by the flow cytometry and western blotting. In flow cytometry, FTVII-modified and unmodified BMSCs were incubated with APC-conjugated anti-mouse HECA452 (BD Biosciences, New Jersey, USA) and APC-conjugated anti mouse CD44 (BioLegend, Beijing, China) for 30 min at 4 °C. The cells were washed twice with PBS, fixed with 2% paraformaldehyde, and finally tested by flow cytometry. In western blotting, the cell lysate proteins were separated by 10% SDS-PAGE electrophoresis gel and then transferred onto polyscreen polyvinylidene difluoride (PVDF) membranes. For detection of sLe^X^, membranes were incubated with mouse HECA452 mAb (NB100-78,039, Novus, Littleton, Colorado, USA), followed by staining with goat anti-rat IgM-HRP (Abcam, Cambridge, UK). The Glyceraldehyde-3-phosphate dehydrogenase (GAPDH) protein level was used as the protein loading control.

### Diabetic mice models

The 8-week-old mice were randomly divided into a normal control group and a diabetic mellitus (DM) group. The mice were fasted overnight and the diabetic models were induced by a single intraperitoneal injection of streptozotocin (STZ) (150 mg/kg; Sigma, MissouriState, USA). STZ was configured in 0.1 mol/L citric acid buffer. The control group mice were given the same volume of 0.1 mol/L citric acid buffer. Blood glucose was measured one week after STZ administration, and mice with blood glucose above 16.7 mmol/L were selected as the diabetic group for the subsequent experiments. Body weight and blood glucose changes were monitored regularly. At the first and eighth weeks of successfully establishing the diabetic models, STZ-induced diabetic mice showed severe hyperglycemia (blood glucose levels ≥ 16.7 mmol/L) compared with the control group mice (*P* < 0.001) and significant reductions in body weight (*P* < 0.001) (Additional file 1: Fig. S1A, B). In addition, compared with the control group, the expression of E-selectin protein (*P* < 0.01, Additional file 1: Fig. S1C) was significantly upregulated, and CD31 staining showed a significant increase in CD31 expression in the colon tissue of mice in the DM group (*P* < 0.01, Additional file 1: Fig. S2A–C).

**Fig. 1:**
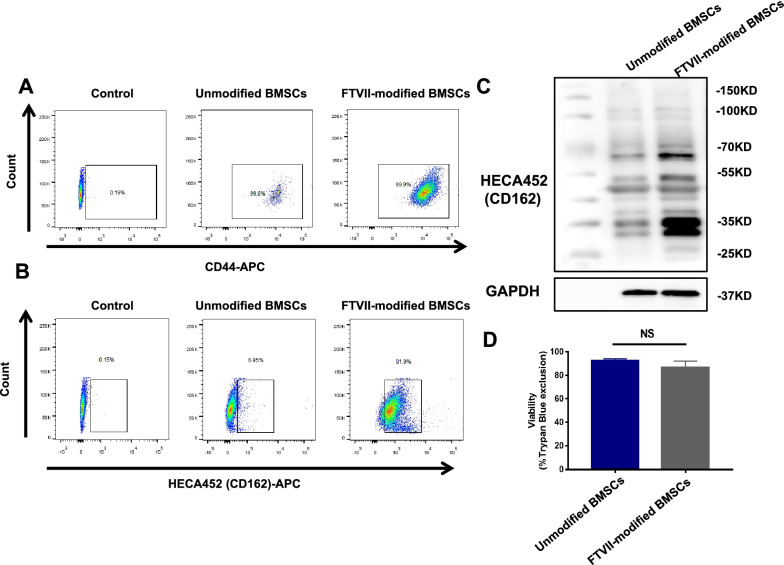
FTVII-mediated α(1,3)-exofucosylation of BMSCs. **A** The expression of CD44 on the surface of FTVII-unmodified and FTVII-modified BMSCs was detected by flow cytometry with APC-conjugated anti-CD44 antibody. **B** The efficiency of exofucosylation modification on BMSCs was evaluated by the reactivity with APC-conjugated anti-HECA452 antibody by the flow cytometry. The result showed that sLe^X^ was hardly expressed in FTVII-unmodified BMSCs, but was highly expressed in the FTVII-modified BMSCs. **C** Western blotting analysis of HECA452 expression of FTVII-unmodified BMSCs and FTVII-modified BMSCs, the positive bands were at  ~ 70KD and ~ 35KD. GAPDH was used as a protein loading control. **D** The cell viability test of unmodified BMSCs and FTVII-modified BMSCs by trypan blue staining. FTVII: fucosyltransferase VII; Control: the isotype control group; Unmodified BMSCs: BMSCs without fucosylation modification; FTVII-modified BMSCs: BMSCs modified with fucosyltransferase VII; CD44-APC: APC-conjugated anti mouse CD44; HECA452-APC: APC-conjugated anti-mouse HECA452

**Fig. 2:**
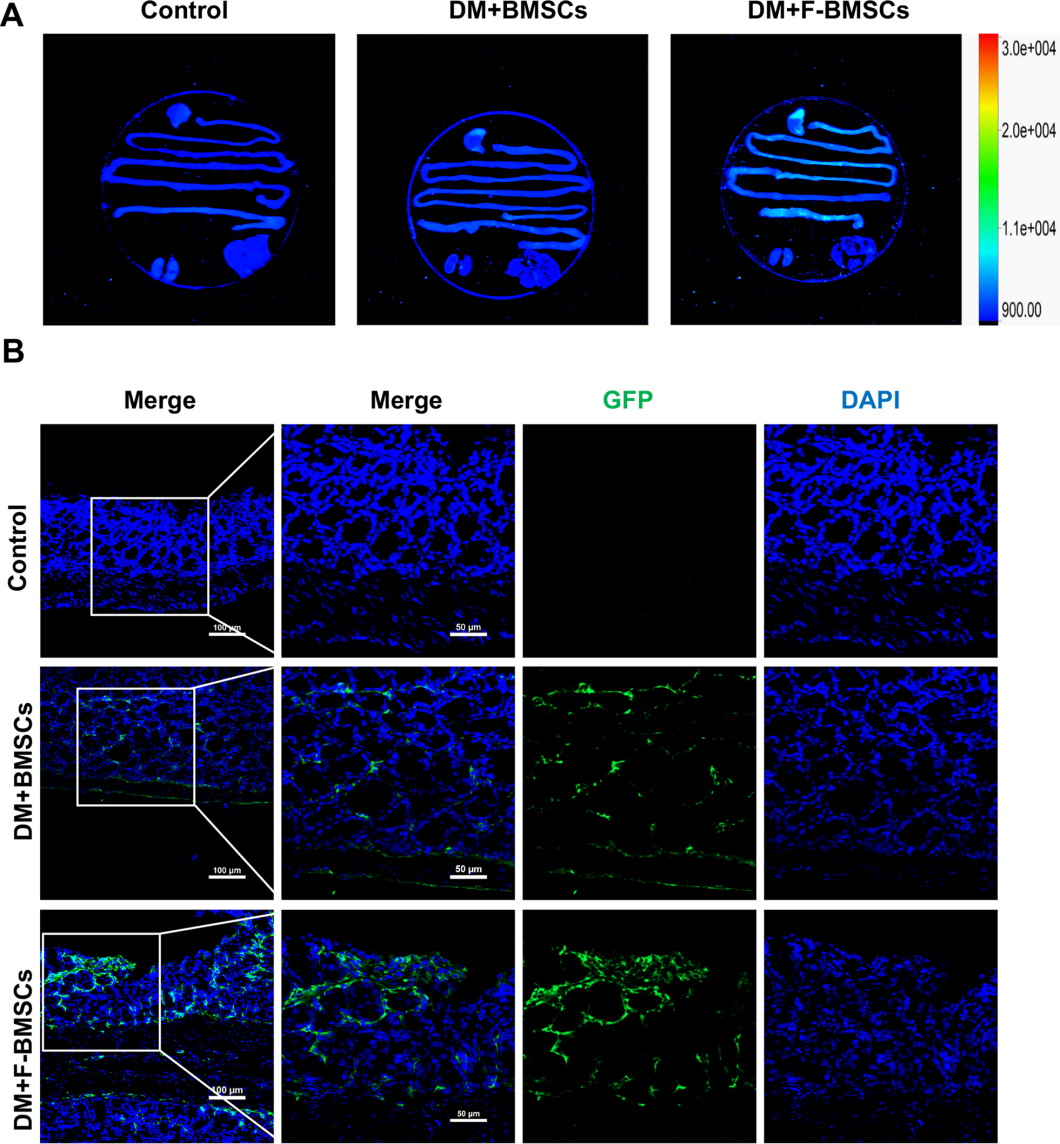
Fucosylation modification enhanced the early migration of BMSCs to the gastrointestinal tract. **A** The bioluminescent imaging results showed that the migration of GFP-labeled FTVII-modified BMSCs to the colon was more than that of unmodified BMSCs. **B** GFP-labeled FTVII-modified BMSCs were mainly distributed in the colonic mucosa, the nuclei (blue); GFP (green). FTVII: fucosyltransferase VII; Control: the control C57 mice; DM + BMSCs: diabetic mice injected with unmodified BMSCs; DM + F-BMSCs: diabetic mice injected with FTVII-modified BMSCs

### Grouping and BMSCs tail vein transplantation

Mice were randomly divided into four groups: control group, DM group, DM mice injected with unmodified BMSCs (DM + BMSCs) group, and DM mice injected with FTVII-modified BMSCs (DM + F-BMSCs) group. Eight weeks after the successful establishment of the diabetic model, FTVII-modified or unmodified BMSCs labeled with GFP were injected into diabetic mice through the tail vein in this study. FTVII-modified or unmodified BMSCs were preconditioned by neurotrophic factor, including glial cell line-derived neurotrophic factor (GDNF), basic-fibroblast growth factor (b-FGF), and epidermal growth factor (EGF) (10 ng/ml; Peprotech, New Jersey, USA) for 10 days before caudal vein transplantation. Follow-up experiments were carried out after eight weeks.

### Short-term migration experiment

The purpose of this experiment was to evaluate the efficiency of FTVII-modified BMSCs on early directional migration to the gastrointestinal nerve injury area. FTVII-modified and unmodified BMSCs labeled with GFP were intravenously injected into DM mice, respectively. The mice were sacrificed 24 h after the injection, and the bioluminescent imaging of gastrointestinal tissues were detected by using a multispectral luminescence system (Bruker MS FX Pro imaging system, Bruker Corp, Billerica, Germany). Then the locations of GFP positive cells in 5-μm frozen sections were detected by confocal laser microscopy (Nikon A1R SI Confocal, Tokyo, Japan).

### Gastrointestinal transmission function detection

To detect the gastrointestinal transmission function, the mice were fasted overnight but had free access to water. The next day each mouse was given 200 μl carbon black suspension (5% powdered carbon suspended in 10% gum arabic) gavage, placed in cages and allowed to move freely. Gastrointestinal transient time was determined as the time period between the gavage and the appearance of the first black fecal pellet. The mice were fed freely, and the frequency of defecation was evaluated by counting the feces per hour. Meanwhile, the feces were collected to measure the total wet weight, and then the dry weight was measured after drying at 100 °C for 2 h. In addition, the stool water content [(Wet weight (mg)—dry weight (mg)/wet weight (mg)] × 100% was calculated.

### Intestinal contractility studies

The colon tissues of each group were cut along the mesentery, then the contents in the cavity were cleaned with Kreb’s solution (NaCl 118.1 mmol/L, KCl 4.8 mmol/L, NaHCO_3_ 25 mmol/L, NaH_2_PO_4_ 1.0 mmol/L, MgSO_4_ 1.2 mmol/L, glucose 11.1 mmol/L, CaCl_2_ 2.5 mmol/L, pH7. 3–7.4, 95% O_2_, 5% CO_2_), and the mucosa and submucosa were removed gently with forceps. Then, the muscle strips (8 mm × 2 mm) were prepared. Both ends of the muscle strips were ligated with fine silk threads and suspended longitudinally in a tissue bath filled with 25 mL Kreb’s solution (37℃, 95% O_2,_ 5% CO_2_). The two ends of the muscle strips were fixed respectively between the water bath and the isometric force sensor (ADInstruments, Dunedin, NewZealand), and then the strips were given an initial load of 1.0 g. After the spontaneous contraction of the muscle strips was stable for 60 min, the contractility (contraction amplitude, frequency and muscle tension) was recorded and analyzed by the polygraph (LabChartsoftwarev.7.0). The muscles strips were then stimulated with 20 V pulses at a frequency of 64 Hz for 10 s to observe their response to electric field stimulation (EFS).

### Immunofluorescence staining

To investigate the morphological changes of myenteric plexus, the muscularis tissues were separated. First, the freshly isolated colon tissues in four groups were placed into the pre-cooled Kreb’s solution, with the mucosa side facing upwards. Then, the mucosa and submucosa were gently torn off with micro tweezers to expose the muscularis tissues, which was further fixed with 4% paraformaldehyde for 10 min. After washing with PBS, the tissues were placed in donkey serum containing 0.3% Triton X-100 and blocked for 24 h to remove the non-specific binding sites. Then, they were incubated with the primary antibody [Rabbit anti-Protein Gene Product 9.5 (PGP9.5) antibody, Abclonal, Wuhan, China; Rabbit anti-Glial Fibrillary Acidic Protein (GFAP) antibody, Abclonal, Wuhan, China; Goat anti-GFP antibody, abcam, Cambridge, UK] containing 0.3% Triton X-100 at 4 °C for 48 h. After washing, tissues were incubated with the secondary antibody (DyLight 594 and donkey anti-rabbit IgG, AntGene, Wuhan, China; DyLight 488 and Donkey anti-mouse IgG, AntGene, Wuhan, China) at 37℃ in the dark for 2 h. Finally, DAPI was used to dye the nucleus for 10 min. On the other hand, in order to observe the expression of PGP9.5, GFAP, and CD31 in a cross-section of the colon, the tissues were immobilized with 4% paraformaldehyde, embedded in paraffin, prepared into sections, dewaxed, followed by serum sealing successively after antigen retrieval, incubated with primary antibody and secondary antibody, and stained with DAPI. Finally, all specimens were observed under the confocal microscopes.

### Western blotting

The colon tissues of mice in each group were lysed in 200 μl Radio Immunoprecipitation Assay (RIPA) lysis buffer (Beyotime, Shanghai, China) and 2μl protease inhibitor (MedChemExpress, New Jersey, USA). The mixture was centrifuged at 12,000 rpm for 15 min, and the supernatants were collected as the total protein extraction. Then the protein concentration was determined by the bicinchoninic acid method. Next, the same amounts of extracted proteins were separated by 12.5% sodium dodecyl sulfate–polyacrylamide gel electrophoresis and transferred on to polyvinylidene fluoride (PVDF) membranes. After being blocked in 10% skim milk at 37℃ for 1 h, the membranes were incubated with the primary antibody (Rabbit anti-PGP9.5 antibody; Rabbit anti-GFAP antibody; Rabbit anti-GDNF antibody, Abclonal, Wuhan, China; Rabbit anti-CD62E/E-selectin antibody, abcam, UK) overnight at 4 °C, and the rabbit anti-GAPDH antibody (Antgene, Wuhan, China) was used as an internal reference control. After washing with TBST 3 times, the membranes were incubated with goat anti-rabbit antibody (AntGene, Wuhan, China) at 37℃ for 1 h. Blots were finally visualized by using the Chemiluminescence imaging system (UVP, USA).

### Statistical analysis

GraphPad Prism v6.0c (GraphPad Software, San Diego, CA, USA) was used for statistical analysis of all experimental data, which were expressed in the form of mean ± standard deviation of at least three independent experiments. Differences between two groups were compared using t-test, differences between multiple groups were compared using one-way ANOVA. *P* value < 0.05 was considered statistically significant.

## Results

### FTVII-mediated α(1,3)-exofucosylation of BMSCs converted cell surface CD44 into HCELL

The flow cytometry data showed that CD44 was highly expressed in BMSCs, while BMSCs did not natively express E-selectin ligand (Fig. 1A, B). In vitro culture, FTVII-mediated α (1,3)-exofucosylation of BMSCs can generate a HCELL property, a fucosylated sialyllactosaminyl glycovariant of CD44 that potently binds E-selectin, as detected by the flow cytometry (Fig. 1B) and western blotting (Fig. 1C). The molecular weights of HECA452 (CD162/HCELL) were predominantly at  ~ 70kD and ~ 35kD (Fig. 1C). In addition, the trypan blue staining showed that FTVII-mediated α (1,3)-exofucosylation of BMSCs was a mild modification and did not significantly affect the cell viability of BMSCs (*P* > 0.05, Fig. 1D).

### Fucosylation modification enhanced the migration of BMSCs to the gastrointestinal tract

To evaluate the degree of migration of FTVII-modified and unmodified BMSCs to gastrointestinal tract after systemic intravenous administration in diabetic mice, GFP-labelled FTVII-modified and unmodified BMSCs were intravenously injected, respectively. Gastrointestinal organs were extracted for fluorescent imaging and prepared as frozen slides to observe the migration of BMSCs within 24 h after injection. Both the bioluminescent imaging and confocal microscopy showed that compared with unmodified BMSCs, FTVII-modified BMSCs had enhanced homing ability to the gastrointestinal tract, mainly to the colon, 24 h after injection through the tail vein in diabetic mice (Fig. 2A, B).

### Transplantation of FTVII-modified BMSCs promoted gastrointestinal motility recovery in diabetic mice

**Fig. 3:**
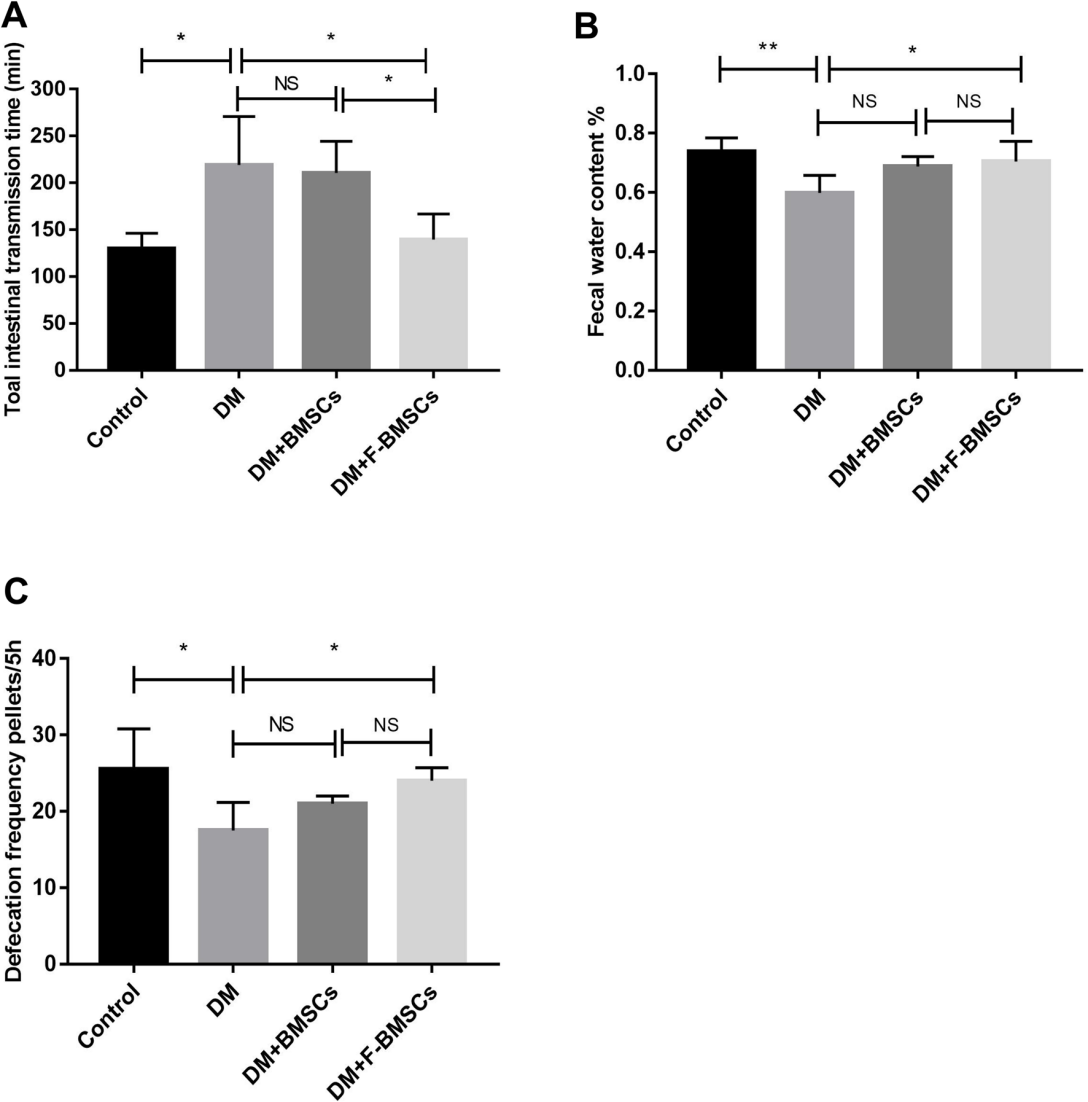
Transplantation of FTVII-modified BMSCs promoted gastrointestinal motility recovery in diabetic mice. The FTVII-modified BMSCs had effects on whole intestinal transit time **(A)**, fecal water content **(B)**, and defecation frenquency **(C).** FTVII: fucosyltransferase VII; Control: the control C57 mice; DM: diabetic mice; DM + BMSCs: diabetic mice injected with unmodified BMSCs; DM + F-BMSCs: diabetic mice injected with FTVII-modified BMSCs. Results were expressed as mean ± SD, *P < 0.05, **P < 0.01, *NS* no significance, n = 6

After intravenous transplantation of FTVII-modified BMSCs or unmodified BMSCs, the changes of gastrointestinal motility in mice were shown in Fig. 3. Compared with the control group, total intestinal transmission time of mice in the DM group was significantly prolonged (*P* < 0.05, Fig. 3A), and the feces moisture content (*P* < 0.01, Fig. 3B) and the defecation frequency was significantly decreased (*P* < 0.05, Fig. 3C). Unmodified BMSCs had no significant effect on the recovery of gastrointestinal motility in diabetic mice (*P* > 0.05, Fig. 3), while FTVII modified BMSCs significantly promoted the gastrointestinal motility recovery (*P* < 0.05, Fig. 3A, B) in DM mice, including reducing the prolonged intestinal transmission time (*P* < 0.05, Fig. 3A), and increasing the water content of the feces (*P* < 0.05, Fig. 3B) and defecation frequency (*P* < 0.05, Fig. 3C). Compared with unmodified BMSCs, FTVII-modified BMSCs significantly restored the intestinal transmission time (*P* < 0.05, Fig. 3A), but not the water content of the feces (*P* > 0.05, Fig. 3B) and defecation frequency (*P* > 0.05, Fig. 3C).

### Transplantation of FTVII-modified BMSCs restored the contraction of the colon in diabetic mice

**Fig. 4:**
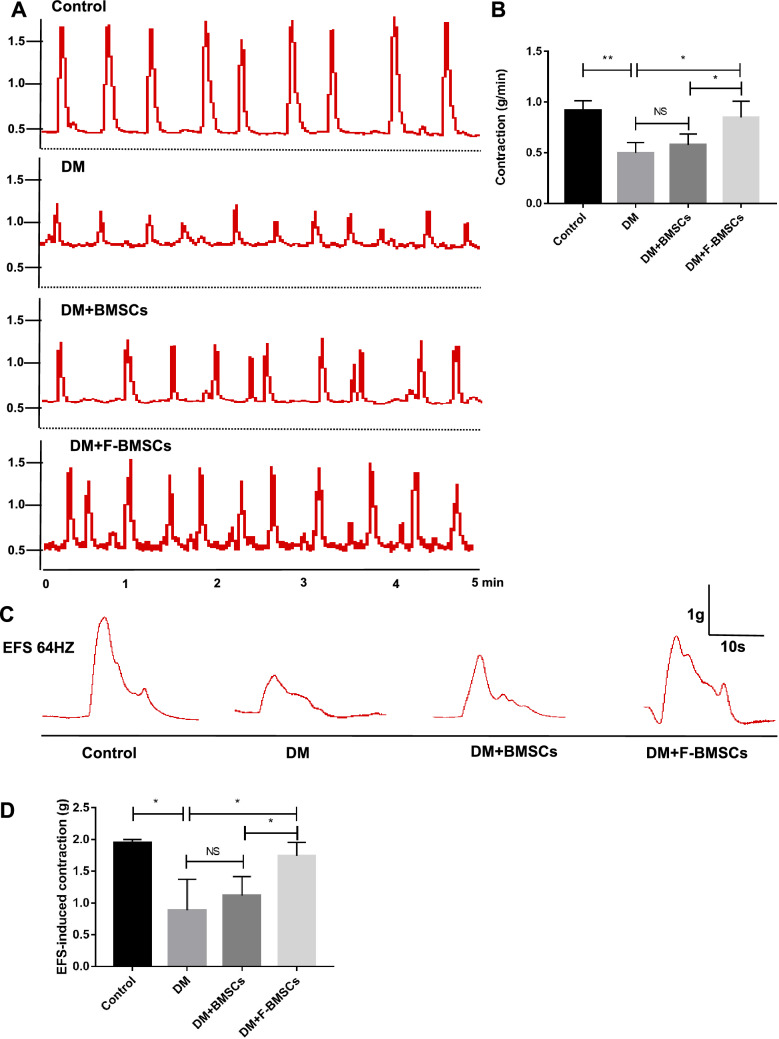
Transplantation of FTVII-modified BMSCs restored the spontaneous contraction of the colon in diabetic mice. **A** The representative schematic diagram of the spontaneous contraction of the colon strips. **B** The contractility (included amplitude, frequency and muscle tension, analyzed as grams for 1 min) of each group. **C** The representative schematic diagram of colon contraction response induced by EFS at the frequency of stimulation (64 Hz) in each group. **D** Quantification of colon strip in response to EFS in each group. FTVII: fucosyltransferase VII; Control: the control C57 mice; DM: diabetic mice; DM + BMSCs: diabetic mice injected with unmodified BMSCs; DM + F-BMSCs: diabetic mice injected with FTVII-modified BMSCs; EFS: electrical field stimulation. Results were expressed as mean ± SD, *P < 0.05, **P < 0.01, *NS* no significance, n = 6

Compared with the control group, the spontaneous contractility of colon muscle strips in diabetic mice was significantly decreased (*P* < 0.01, Fig. 4A, B), and the contractile response of colon muscle strips induced by EFS was severely impaired (*P* < 0.05, Fig. 4C, D). Then, we evaluated the effects of FTVII-modified and unmodified BMSCs on the spontaneous contraction and the contractile response of colon muscle strips in diabetic mice. Compared with unmodified BMSCs, FTVII-modified BMSCs had a significant restoration effect on colonic contraction in in diabetic mice (*P* < 0.05, Fig. 4). Correspondingly, unmodified BMSCs had no significant effect on the repair of colonic contraction in diabetic mice (*P* > 0.05, Fig. 4), while FTVII-modified BMSCs significantly improved impaired colonic contraction in DM mice, including spontaneous contractility (*P* < 0.05, Fig. 4A, B) and the contractile response induced by EFS (*P* < 0.05, Fig. 4C, D).

### Effect of FTVII-modified BMSCs on the ENS remodeling in diabetic mice

**Fig. 5:**
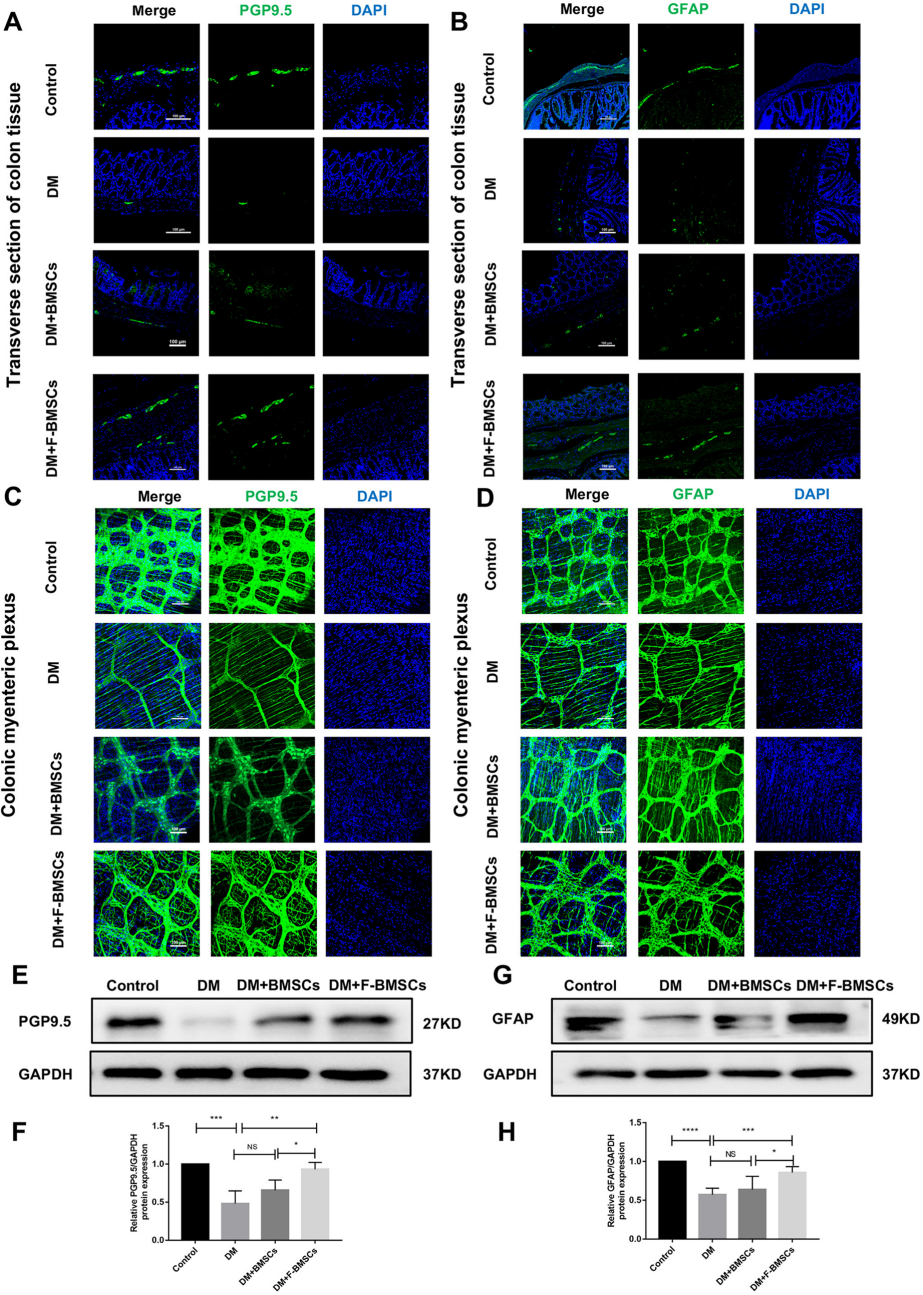
Effect of FTVII-modified BMSCs on the ENS remodeling in diabetic mice. **A** Representative immunofluorescence confocal laser images in transverse sections of colon tissue sections of PGP9.5(green) in each group; the nuclei (blue). **B** Representative immunofluorescence confocal laser images in transverse sections of colon tissue sections of GFAP (green) in each group; the nuclei (blue); **C** Representative immunofluorescence confocal laser images in colon myenteric plexus of PGP9.5 (green) in each group. **D** Representative immunofluorescence confocal laser images in colon myenteric plexus of GFAP (green) in each group. **E** The protein expression of PGP9.5 in the colon tissues was examined by immunoblotting. **F** The statistics results of PGP9.5 protein expression in the colon tissues. **G** The protein expression of GFAP in the colon tissues was examined by immunoblotting. **H** The statistics results of GFAP protein expression in the colon tissues. Control: the control C57 mice; DM: diabetic mice; DM + BMSCs: diabetic mice injected with unmodified BMSCs; DM + F-BMSCs: diabetic mice injected with FTVII-modified BMSCs. These results are representative of at least three times independent experiments. Results were expressed as mean ± SD,*P < 0.05,**P < 0.01, ***P < 0.001, ****P < 0.001, *NS* no significance

Immunofluorescence detection in the slides revealed that PGP9.5 and GFAP were mainly distributed in the myenteric plexus of the colon (Fig. 5A, B). At the 8th week of the course of diabetes, compared with the control group, the expression of PGP9.5 (*P* < 0.01, Fig. 5A、C and Additional file 1: Fig. S1D) and GFAP (*P* < 0.01, Fig. 5B, D and Additional file 1: Fig. S1E) in the myenteric plexus of the colon were decreased, and the sizes of neurons and glial cells were smaller (Fig. 5A–D). Eight weeks after transplantation of FTVII-modified BMSCs into diabetic mice, the expressions of PGP9.5 and GFAP were significantly increased (*P* < 0.05, *P* < 0.0001, Fig. 5A-D, and Additional file 1: Fig. S1D–E), and the number of neurons and glial cells increased, their size returned to normal, and their arrangement became regular (Fig. 5A-D); whereas transplantation of unmodified BMSCs into diabetic mice had no significant effect (*P* > 0.05). Moreover, the western blotting also confirmed that FTVII-modified BMSCs significantly upregulated the protein expression levels of PGP9.5 (*P* < 0.01) and GFAP (*P* < 0.001) when compared with the diabetic mice, while unmodified BMSCs did not have a significant effect (*P* > 0.05, Fig. 5E–H). In addition, compared with unmodified BMSCs, FTVII-modified BMSCs significantly upregulated the protein expressions of PGP9.5 and GFAP (*P* < 0.05, Fig. 5E–H).

**Fig. 6:**
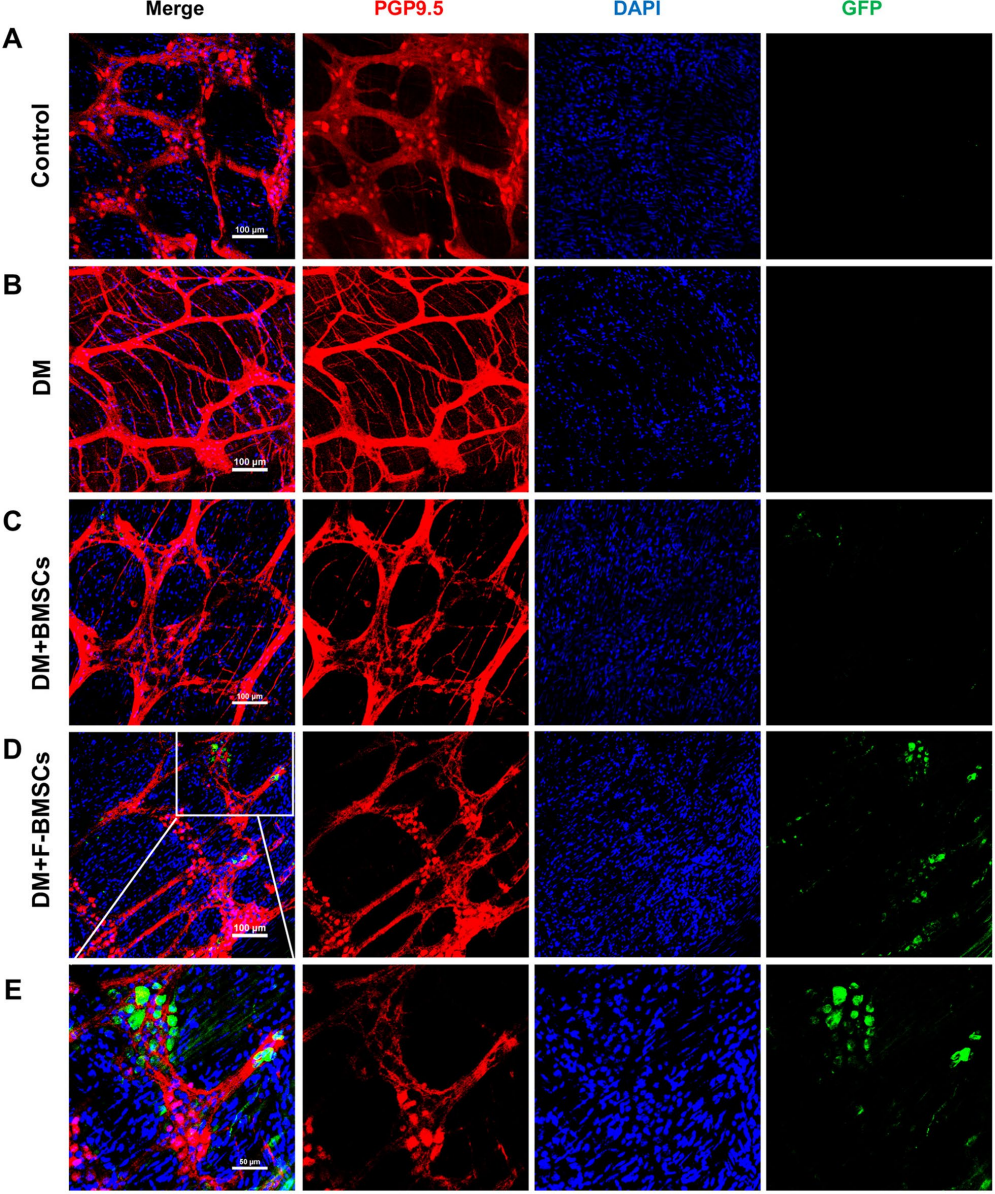
Effect of FTVII-modified BMSCs on the ENS remodeling in diabetic mice. **A–D** GFP-labeled unmodified and FTVII-modified BMSCs (green) and PGP9.5 (red) were jointly immunostained in the freshly intermuscular plexus tissues of colon. The nuclei were labeled with DAPI (blue). **E** The partial enlargement images of the DM + F-BMSCs group. Control: the control C57 mice; DM: diabetic mice; DM + BMSCs: diabetic mice injected with unmodified BMSCs; DM + F-BMSCs: diabetic mice injected with FTVII-modified BMSCs

**Fig. 7:**
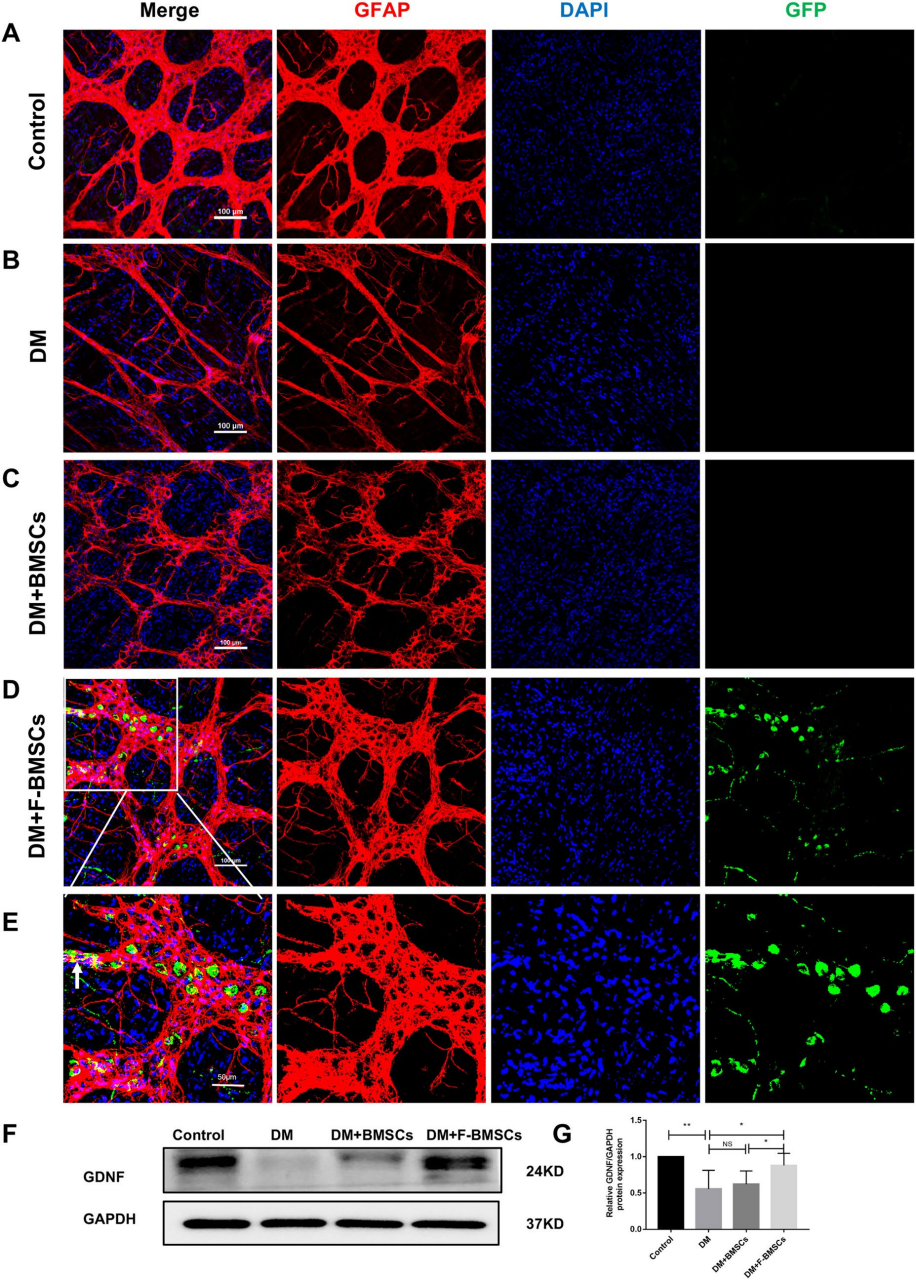
Effect of FTVII-modified BMSCs on the ENS remodeling in diabetic mice. **A–D** GFP-labeled unmodified and FTVII-modified BMSCs (green) and GFAP (red) were jointly immunostained in the freshly intermuscular plexus tissues of colon. The nuclei were labeled with DAPI (blue). **E** The partial enlargement images of the DM + F-BMSCs group. **F** The protein expression of GDNF in the colon tissues was examined by immunoblotting. **G** The statistics result of GDNF protein expression in the colon tissues. Control: the control C57 mice; DM: diabetic mice; DM + BMSCs: diabetic mice injected with unmodified BMSCs; DM + F-BMSCs: diabetic mice injected with FTVII-modified BMSCs. These results are representative of at least three times independent experiments. Results were expressed as mean ± SD, *P < 0.05, **P < 0.01, *NS* no significance

At 8 weeks after BMSCs transplantation into diabetic mice, fresh colon muscle plexus tissues were performed to trace GFP-labeled BMSCs (Figs. 6 and 7), and FTVII-modified BMSCs were found in the intermuscular nerve plexus (Fig. 6D–E, 7D–E), suggesting that they could migrate to the area of ENS injury and survive for a prolonged duration. Immunostaining of GFP-labeled FTVII-modified BMSCs in combination with PGP9.5 or GFAP showed that BMSCs did not present as PGP9.5-positive cells, but surrounded PGP9.5-positive cells, and could show positive GFAP expression in fresh colon muscle plexus tissue (Figs. 6D–E and 7D–E). In addition, the protein expression level of GDNF in the colon tissues was down-regulated more significantly in the DM group than the control group (*P* < 0.01, Fig. 7F–G). Further, FTVII-modified BMSCs significantly upregulated the protein expression levels of GDNF (*P* < 0.05, Fig. 7F–G) when compared with the diabetic mice, while unmodified BMSCs had no impact (*P* > 0.05, Fig. 7F–G). In addition, compared with unmodified BMSCs, FTVII-modified BMSCs significantly upregulated the protein expressions of GDNF (*P* < 0.05, Fig. 7F–G).

## Discussion

Studies have shown that BMSCs can treat diabetic peripheral neuropathy [12, 26, 27], but whether they can treat diabetes-related ENS damage remains unclear. This study demonstrated for the first time that fucosylation modified-BMSCs transplanted into mice through the tail vein could promote the remodeling of colonic myenteric neurons and glial cells, thus promoting gastrointestinal motility.

Previous studies showed that total neurons in the myenteric plexus of the gastrointestinal tract of diabetic rats were significantly reduced [28, 29]. In addition, Liu et al. found that the expression level of GFAP in diabetic rats’ colon tissues decreased at 4 weeks after STZ injection, and decreased more significantly at 12 weeks [30]. Consistent with previous studies, our results found that PGP9.5 and GFAP decreased in colon tissues of diabetic mice induced by STZ at 16 weeks. Diabetic colonic motility disorder is characterized by abdominal distension and constipation, and its pathophysiological characteristics include decreased colonic tension and contractility, slow peristalsis, and delayed emptying [7, 31–33]. Moreover, in our study, we also found that compared with normal mice, the autonomic contractile ability of colonic muscles and the ability to respond to electric field stimulation in diabetic mice were reduced, the intestinal transmission ability was poor, and the fecal water content and frequency of defecation were reduced.

BMSCs show great potential for application in tissue regeneration engineering and cell transplantation treatment for various diseases due to their immunomodulatory properties, safety and strong in vitro expansion ability [12, 34]. However, one of the central obstacles in BMSCs cell therapy is the delivery of BMSCs to the target tissue. Due to the wide range of ENS lesions in diabetic mice, local transplantation of BMSCs is difficult to achieve feasibility and effectiveness in clinical applications. Theoretically, BMSCs can migrate to the entire gastrointestinal tract by vein transplantation, but it is typically difficult for ordinary BMSCs to reach to the damaged sites after intravenous transplantation. Therefore, it is necessary to improve its directional homing ability. The homing process of BMSCs is similar to the exudation process of a large number of white blood cells after local inflammation in the human body, including the processes of aggregation and rolling, adhesion and migration [16, 35]. In this study, to promote the aggregation and rolling of BMSCs to enhance their homing ability and thus optimize ENS regeneration, we attempted to increase the binding of BMSCs to E-selectin on the surface of vascular endothelial cells through HCELL expression. Reza et al. found that the number of cells migrating to pancreatic islets after tail vein injection of fucosylation modified-BMSCs in diabetic mice was three times that of unmodified BMSCs, with significantly improved efficacy in reversing autoimmune diabetes [18]. Our results showed that the expression of E-selectin protein in the colon tissue of diabetic mice was higher than that of normal mice and the migration of fucosylation modified-BMSCs into the colon of diabetic mice could be observed 24 h after caudal vein transplantation, while the migration number of BMSCs without fucosylation modification was less. Based on our results, the increased migration ability of BMSCs should be considered foundational in the treatment of intestine nerve injury.

Previous reports have demonstrated that BMSCs have great potential in the treatment of gastrointestinal motility disorders. Mazzanti et al. found that use of local injection of autologous BMSCs could improve the regeneration of the LES sphincter after esophagogastric incision in rats with achalasia, and increase muscle contractility, which was beneficial to prevent gastroesophageal reflux symptoms [36]. Ishii et al. showed that tail vein transplantation of bone marrow cells could promote gastrointestinal motility disorders of ICC-deficient mice by differentiating into ICC [37]. Further, our previous study demonstrated that BMSCs could improve muscle stripe contraction and electric field stimulation response with local ENS injury of the stomach in rats [25]. Similarly, in this study, compared with unmodified BMSCs, fucosylation modified-BMSCs were shown to have significant effects on the recovery of intestinal dynamics and ENS remodeling after colonic nerve injury in diabetic mice, including intestinal transport time, expression of glial cell marker proteins and expression of neuronal marker proteins, but had no significant effects on fecal water content and defecation frequency.

Diabetic neuropathy manifests as a deficiency in peripheral nerves and blood vessels, and a lack of angiogenic factors and neurotrophic factors [12]. GDNF is a neurotrophic factor that has nutritional and protective effects on injured nerve cells. Du et al. found that compared with normal rats, the expression levels of GDNF in the colon of diabetic rats were significantly down-regulated [38]. It has also been found that the concentration of GDNF in the serum of patients with type 2 diabetes is significantly reduced compared with those with normal glucose tolerance [39]. These combined findings suggest that the occurrence of diabetic gastrointestinal nerve damage may be related to the decreased expression of GDNF. Our previous study found that orthotopic transplantation of preconditioned BMSCs in the ENS deletion site mainly promoted the repair of ENS by secreting GDNF [25]. Moreover, Ezquer et al. found that injecting fat-derived MSCs into the vitreous of STZ-induced diabetic mice with diabetic retinal damage could prevent the loss of retinal ganglion cells by increasing the levels of various neurotrophic factors in the eye and alleviating the oxidative damage of the retina [40]. Correspondingly, in this study, we found that compared with normal mice, the expression level of GDNF in the colon tissue of diabetic mice was significantly decreased. After transplantation of FTVII-modified BMSCs, the GDNF protein level was up-regulated, and both neuronal and glial cell markers were increased. In addition, we also found that preconditioned fucosylated FTVII-modified BMSCs could migrate to the myenteric plexus of colon and partially differentiated to GFAP. The up-regulated GDNF protein level in suffering colons may partially come from BMSCs, or from recovered glial cells.

Furthermore, our study demonstrated that the fucosylation on the surface of BMSCs was a mild modification without changing its activity, thus providing feasibility and great potential for its clinical application. Diabetic gastrointestinal motility disorder has a high clinical incidence, but currently there is a lack of effective treatment methods. We explored fucosylation modified BMSCs to optimize ENS remodeling and restore intestinal motility, providing a basis for cell therapy for enteric neuropathy.

## Conclusions

In conclusion, the present study has demonstrated that FTVII-modified BMSCs can effectively migrate to the colon of diabetic mice through caudal vein transplantation, and can facilitate the recovery of gastrointestinal dynamics of diabetic mice by promoting the remodeling of ENS.

## Supplementary Information

**Additional file 1: Figure S1.** Body weight (**A**) and blood glucose levels (**B**) in mice. **C** The expression level of E-selectin protein in Control and DM group. These results are representative of at least three times independent experiments. **D–E** The statistics results of PGP9.5 and GFAP positive staining ratio to entire colonic mucosa sections. Control: the control C57 mice; DM: diabetic mice. Results were expressed as mean ± SD,*P < 0.05,**P < 0.01, ****P < 0.0001, NS: no significance. **Figure S2. A–B** Representative immunofluorescence confocal laser images in sections of CD31 (red) and the nuclei (blue) in Control and DM mice. **C** The statistics result of CD31 positive staining ratio to entire colonic mucosa sections. Control: the control C57 mice; DM: diabetic mice. Results were expressed as mean ± SD, **P < 0.01.

## Data Availability

The datasets used and/or analyzed during the current study are available from the corresponding author on reasonable request.

## Abbreviations

BMSCs: Bone marrow-derived mesenchymal stem cells
b-FGF: Basic-fibroblast growth factor
DM: Diabetic mellitus
DMEM: Dulbecco's modified eagle's modified medium
ENS: Enteric nervous system
EFS: Electric field stimulation
EGF: Epidermal growth factor
FTVII: Fucosyltransferase VII
GFP: Green fluorescent protein
GFAP: Glial fibrillary acidic protein
GAPDH: Glyceraldehyde-3-phosphate dehydrogenase
GDNF: Glial cell line-derived neurotrophic factor
HBSS: Hanks balanced salt solution
HSA: Human serum albumin
PBS: Phosphate-buffered saline
PVDF: Polyscreen polyvinylidene difluoride
PGP9.5: Protein Gene Product 9.5
sLe^X^: Sialyl-Lewis X
STZ: Streptozotocin
