# Supplementary figures and images for: CD44 fucosylation on bone marrow-derived mesenchymal stem cells enhances homing and promotes enteric nervous system remodeling in diabetic mice
Source: Cell Biosci. 2021 Jun 30;11:118. doi: 10.1186/s13578-021-00632-2 (PMC8243650; doi:10.1186/s13578-021-00632-2)

# Supplementary Figure 1

**A**

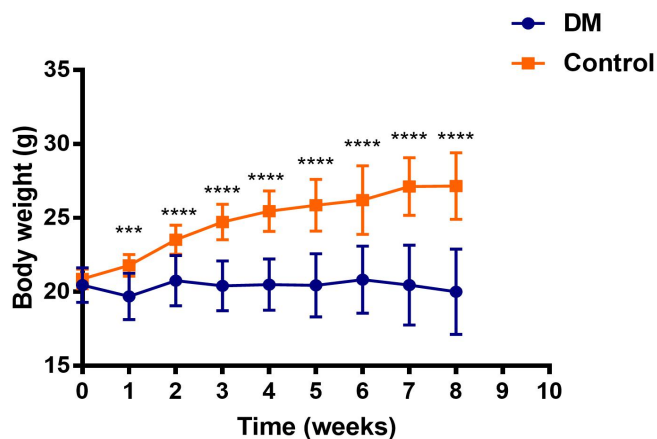

**B**

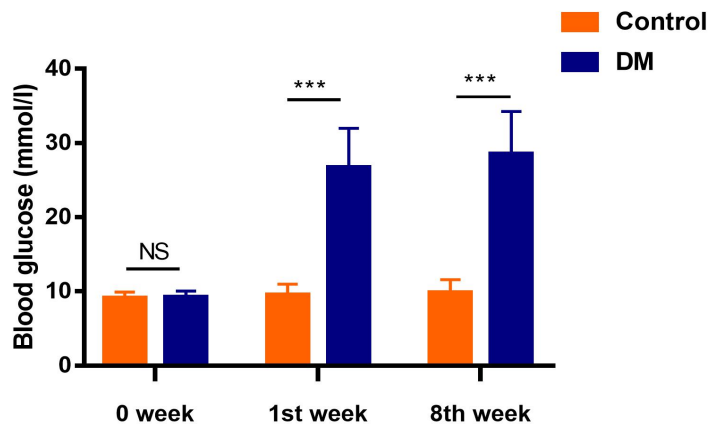

**C**

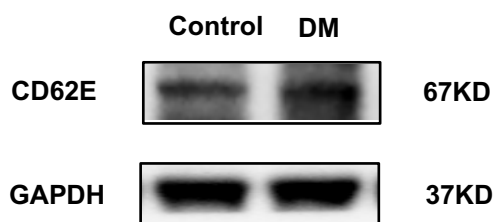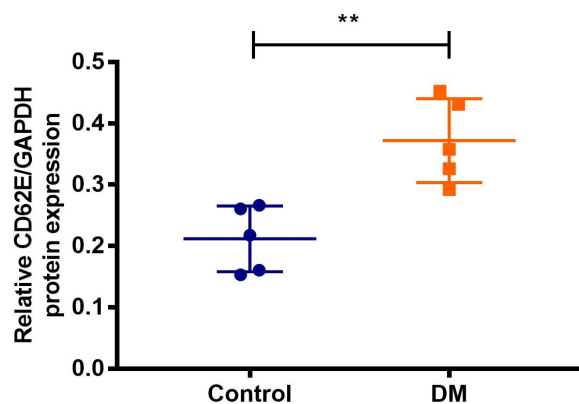

**D**

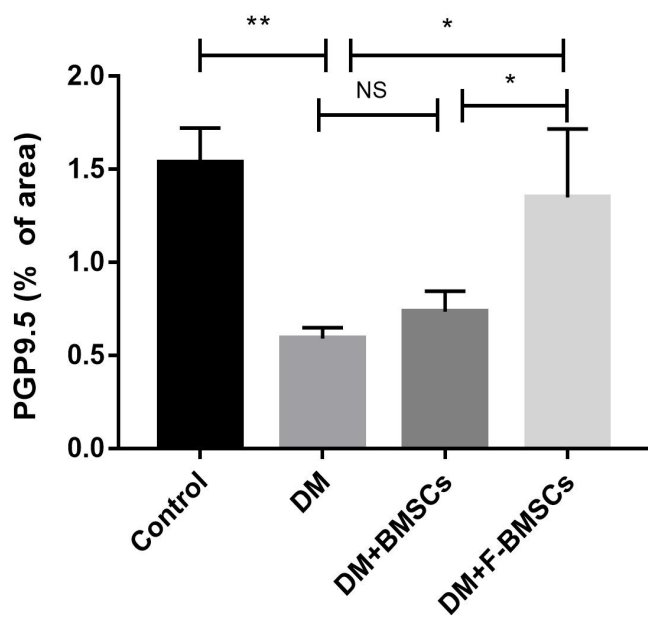

**E**

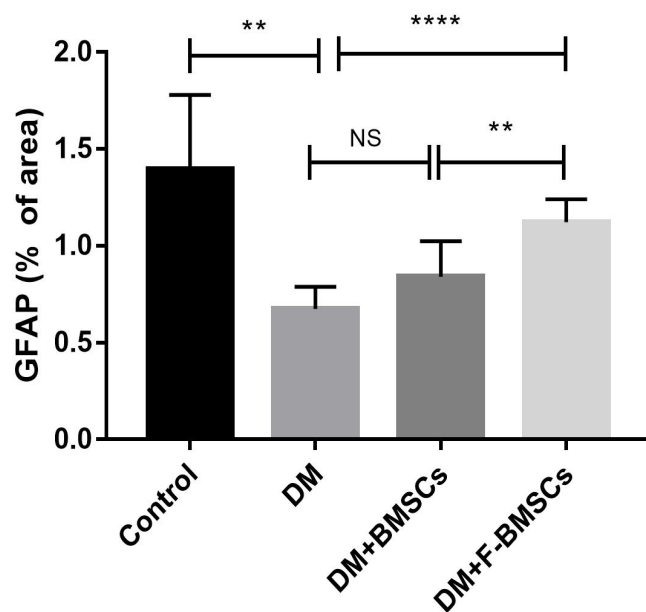

Supplementary Figure 2

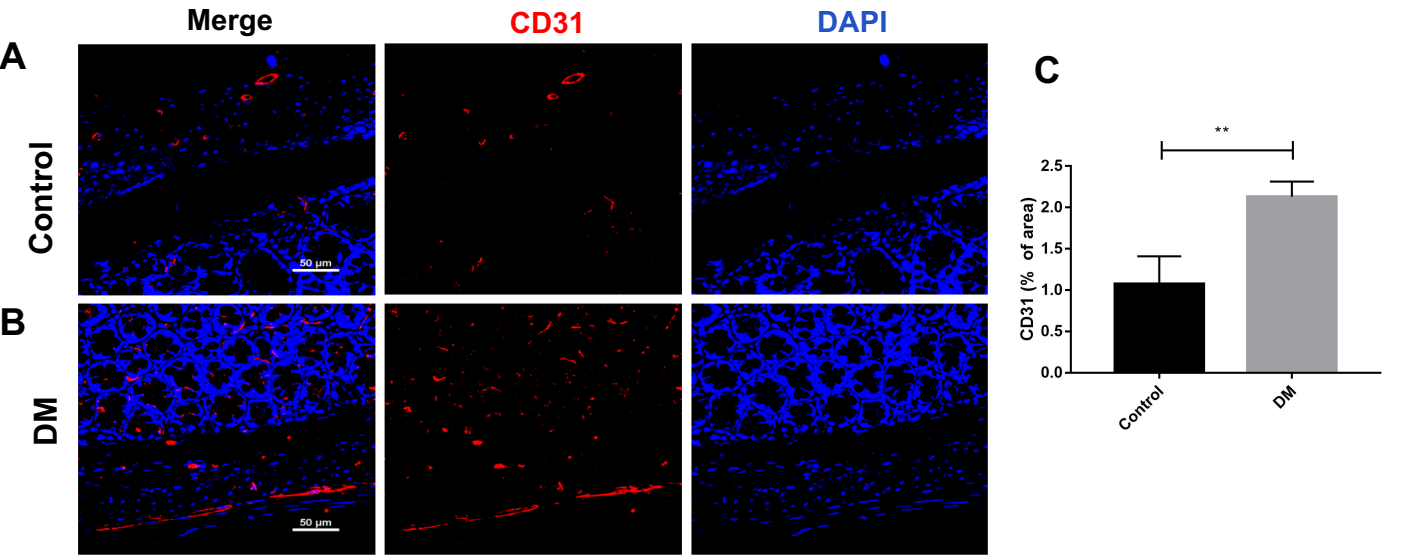

Supplement: Supplementary file 1 — Additional file 1: Figure S1. Body weight (A) and blood glucose levels (B) in mice. C The expression level of E-selectin protein in Control and DM group. These results are representative of at least three times independent experiments. D–E The statistics results of PGP9.5 and GFAP positive staining ratio to entire colonic mucosa sections. Control: the control C57 mice; DM: diabetic mice. Results were expressed as mean ± SD,*P < 0.05,**P < 0.01, ****P < 0.0001, NS: no significance. Figure S2. A–B Representative immunofluorescence confocal laser images in sections of CD31 (red) and the nuclei (blue) in Control and DM mice. C The statistics result of CD31 positive staining ratio to entire colonic mucosa sections. Control: the control C57 mice; DM: diabetic mice. Results were expressed as mean ± SD, **P < 0.01. [file 13578_2021_632_MOESM1_ESM.pdf]
